# Supplementary material for: Characterization of Euglena gracilis Mutants Generated by Long-Term Serial Treatment with a Low Concentration of Ethyl Methanesulfonate
Source: Microorganisms. 2025 Feb 8;13(2):370. doi: 10.3390/microorganisms13020370 (PMC11858117; doi:10.3390/microorganisms13020370)
Supplement: Supplementary file 1 [file microorganisms-13-00370-s001.zip › microorganisms-3451929-supplementary.pdf]

Characterization of *Euglena gracilis* mutants generated by long-term serial treatment with a low concentration of ethyl methanesulfonate

Ji-Yeon Kang, Younglan Ban, Eui-Cheol Shin, Jong-Hee Kwon*

***Corresponding authors:**

Jong-Hee Kwon

E-mail: jhkwon@gnu.ac.kr

Content

Supplemental Table

Supplementary Table S1. Concentrations (μg/100 g) of volatile compounds in *Euglena* WT and *Euglena* Mutant 335 based on GC/MS………………………………………………………………………….....2

Supplementary Table S1. Concentrations (μg/100 g) of volatile compounds in *Euglena* WT and *Euglena* Mutant 335 based on GC/MS

Volatile compound	RT[1] (min)	RI[2]	Mean±SD		I.D.[3]
			Mutant 335	WT (367)	
**Alcohols (19)**					
2,2-Dimethyl-3-(1-octylamino)-4-nonanol	9.25	850	ND[4]	33.09±46.79	MS
1-Hexanol	10.29	879	ND	80.01±113.15	MS
1-Octen-3-ol	13.83	984	ND	362.54±512.71	MS
2-Ethylhexanol	15.39	1032	148.42±91.16	546.36±414.41	MS/RI[5]
2,2-Dimethyl-1-octanol	17.44	1096	ND	102.56±70.91	MS
1-Hexadecanol	18.85	1145	78.01±58.64	50.64±46.75	MS
1-Nonanol	19.64	1172	ND	98.97±139.97	MS
2-Methyl-1-undecanol	20.23	1191	51.70±73.11	ND	MS
1-Decanol	22.43	1271	210.94±134.27	ND	MS
2-Propylheptanol	24.37	1344	ND	10.78±15.25	MS
1-Undecanol	25.06	1370	35.14±49.69	165.37±233.87	MS
Pentadecanol	28.41	1503	123.05±174.02	ND	MS
1-Docosanol	28.45	1505	51.48±72.81	ND	MS
2,5-Bis(1,1-dimethylethyl)-phenol	28.49	1507	ND	78.46±110.96	MS
1-Heptadecanol	29.07	1532	75.86±107.28	ND	MS
Tridecanol	29.92	1568	686.01±970.17	1,342.40±396.45	MS

Isoheptadecanol	31.35	1630	64.10±90.66	57.52±81.35	MS
(E)-3-Nonen-1-ol	31.80	1650	7.95±11.25	ND	MS
1-Octadecanol	33.69	>1700	28.24±39.93	100.94±142.75	MS
**Aldehydes (7)**					
Hexanal	7.99	810	ND	231.23±327.01	MS/RI
2,2-Dideutero heptadecanal	10.04	872	ND	73.52±103.97	MS
Benzaldehyde	13.24	967	ND	106.47±150.57	MS/RI
Tetradecanal	26.00	1406	145.74±206.11	149.41±211.29	MS
3-Methyl-3-cyclohexen-1-carboxaldehyde	28.91	1525	ND	38.06±53.82	MS
Tetradecanal	30.78	1604	107.67±117.11	ND	MS
4-Octadecenal	33.53	>1700	ND	9.68±13.69	MS
**Sulfur-containing compounds (3)**					
Di-tert-dodecyldisulfide	17.81	1108	39.69±56.14	ND	MS
Dihexylsulfide	18.57	1136	ND	4.43±6.27	MS
Benzothiazole	21.26	1228	80.73±34.47	282.74±162.22	MS/RI
**Acids & esters (18)**					
2-Propenoic acid	5.55	<800	ND	525.59±743.30	MS
Methyl methacrylate	5.59	<800	ND	118.93±168.20	MS
Hexyl formate	10.21	877	ND	454.78±643.16	MS
4-Ethylbenzoic acid	11.31	907	ND	393.45±556.42	MS
Sulfurous acid, butyl nonyl ester	16.08	1055	ND	50.38±71.24	MS

Octyl chloroformate	16.68	1073	ND	275.03±388.96	MS
Acetic acid, octyl ester	16.69	1074	ND	96.28±136.16	MS
Methyl salicylate	20.41	1196	ND	236.47±334.42	MS
Decyl ether	20.96	1217	ND	26.67±0.05	MS
Cyclohexyl isothiocyanate	21.49	1237	35.79±50.61	252.89±106.97	MS
Didecyl sebacate	26.97	1446	ND	26.82±37.93	MS
Heptadecyl heptadecanoate	27.95	1485	47.75±67.53	ND	MS
Octadecyl bromoacetate	29.08	1533	ND	32.61±46.12	MS
Chloroacetic acid, octadecyl ester	34.29	>1700	ND	48.87±69.11	MS
Dodecyl fluoroacetate	34.92	>1700	9.68±13.69	ND	MS
Undec-2-enylester dichloroacetic acid	35.09	>1700	6.17±8.73	ND	MS
8,11,14-Eicosatrienoic acid, methyl ester	35.89	>1700	ND	9.89±13.99	MS
Hexadecyl bromoacetate	36.34	>1700	ND	47.08±66.57	MS
**Heterocyclic compounds (6)**					
Pyridine	6.52	<800	ND	180.37±89.56	MS
Pyridiniumfluorosulfate	6.79	<800	ND	6.07±8.58	MS
2-Pentylfuran	14.24	995	ND	340.05±480.91	MS
Camphor	19.00	1150	ND	15.84±22.40	MS/RI
2-Chloro-3,4-diphenylbenzofuro[2,3-b] pyridine	25.34	1381	ND	30.83±43.60	MS
**Hydrocarbons (67)**					

1,2-Bis(trimethylsilyl)-benzene	7.91	807	ND	155.45±219.84	MS
2,4-Dimethylheptane	8.34	821	12.15±17.18	ND	MS
4-Methyloctane	9.78	865	170.19±240.69	392.21±554.66	MS
2,3,4-Trimethylhexane	10.09	874	24.48±34.62	ND	MS
2-Methyl-1-heptene	10.34	881	ND	30.58±43.25	MS
Styrene	10.98	897	ND	201.00±284.26	MS/RI
2,7-Dimethyloctane	12.18	936	ND	43.37±61.34	MS
2,6-Dimethyloctane	12.33	940	ND	31.72±44.85	MS
Decane	13.00	960	473.20±214.07	3,814.51±4,302.46	MS
2-Methylnonane	13.29	969	96.32±136.21	ND	MS
Nonadecane	13.33	970	63.78±15.33	478.92±345.75	MS
3-Methylhexane	13.54	976	ND	134.60±190.35	MS
2,4-Dimethylhexane	13.73	981	ND	243.84±344.85	MS
1-Hexyl-3-methylcyclopentane	14.08	990	92.92±131.41	ND	MS
3,6-Dimethylundecane	14.78	1012	40.20±56.85	288.22±239.30	MS
2,7-Dimethylundecane	14.94	1017	32.06±45.34	184.92±261.51	MS
2,5-Dimethylnonane	15.11	1023	50.27±71.09	521.16±416.32	MS
4-Methyldecane	15.19	1026	202.41±169.29	1,352.80±994.61	MS
Pentyl-cyclopentane	15.61	1040	ND	109.91±155.43	MS
3-Ethyl-5-(2-ethylbutyl)-octadecane	15.75	1044	6.79±9.60	19.85±28.08	MS
Dodecane	16.15	1057	1,230.86±37.26	3,786.44±2624.40	MS
2,6,11-Trimethyldodecane	16.20	1058	ND	53.89±76.21	MS

Octadecane	16.46	1066	107.69±152.30	ND	MS
2,4-Dimethylundecane	16.47	1067	79.67±40.12	80.65±27.23	MS
4-Ethyl-1,2-dimethyl-benzene	17.18	1088	ND	40.07±56.67	MS
Undecane	17.57	1100	23.69±33.50	229.53±85.60	MS/RI
4-Methylundecane	17.64	1102	242.59±59.44	421.89±32.85	MS
Heneicosane	17.81	1109	ND	304.15±304.11	MS
1,2,3,4-Tetramethylbenzene	18.23	1123	ND	41.85±59.18	MS
2-Methylundecane	18.39	1129	ND	123.24±145.29	MS
2,6,10-Trimethyltetradecane	18.57	1135	ND	23.03±32.57	MS
(E)-1-Butyl-2-methylcyclopropane	19.07	1153	ND	16.07±22.73	MS
6-Ethyl-2-methyloctane	19.31	1161	ND	56.13±79.38	MS
Squalane	19.44	1165	ND	54.53±77.12	MS
Cyclododecane	20.24	1191	8,955.69±8,032.29	17,120.77±5,004.40	MS
Tritetracontane	20.61	1204	ND	123.01±173.96	MS
5-Methyloctadecane	20.77	1210	17.02±24.07	55.07±77.87	MS
2,6-Dimethylundecane	20.86	1213	ND	240.77±96.84	MS
2,4-Dimethylicosane	20.96	1217	ND	25.64±36.26	MS
3-Ethyltetracosane	21.08	1221	ND	120.26±170.08	MS
Tetracosane	21.69	1244	ND	140.68±0.59	MS
2,4,6-Trimethyldecane	21.84	1249	39.19±21.94	64.26±90.88	MS
1,3-Bis(1,1-dimethylethyl)-benzene	22.03	1257	181.02±90.18	655.22±220.60	MS
4-Ethylundecane	22.22	1263	51.18±72.38	10.07±14.24	MS

Nonacosane	22.23	1264	ND	126.54±178.95	MS
Tetradecane	22.67	1279	116.96±100.04	1,545.25±1,439.78	MS
1,3-Dichloro-2-methoxybenzene	22.97	1290	ND	16.32±23.09	MS
Tridecane	23.04	1292	18.01±25.47	77.50±109.60	MS/RI
2,3,5,8-Tetramethyldecane	23.18	1297	9.06±12.82	ND	MS
Hexacosane	23.25	1299	ND	32.14±45.45	MS
2,3,5-Trimethyldecane	23.41	1306	ND	35.18±49.75	MS
4-Methyltetradecane	24.31	1341	ND	10.17±14.39	MS
2,6,10-Trimethyltridecane	24.32	1342	ND	6.31±8.93	MS
10-Methylnonadecane	24.82	1361	ND	6.62±9.36	MS
1,1-Bis(dodecyloxy)-hexadecane	26.68	1434	12.28±17.36	87.10±72.05	MS
2-Methyltetradecane	27.29	1459	ND	112.17±21.79	MS
Docosane	28.10	1491	ND	108.51±55.08	MS
1-Chlorooctadecane	28.85	1523	ND	7.81±11.05	MS
3-Methyltridecane	29.15	1535	ND	38.21±54.03	MS
(E)-3-Octadecene	29.25	1540	ND	49.04±69.36	MS
2-Methyloctadecane	29.81	1564	ND	58.60±82.87	MS
Hexadecane	30.46	1591	32.71±46.25	756.99±541.19	MS/RI
2-Methyltetracosane	31.86	1652	ND	21.11±29.86	MS
(E)-2-Tetradecene	32.16	1665	4,124.93±4,333.01	8,371.63±1,937.56	MS
1-Fluorododecane	32.64	1686	61.35±86.77	ND	MS
Cyclotetradecane	34.92	>1700	80.22±113.44	77.86±8.47	MS

1,3-Cyclooctadiene	40.67	>1700	ND	50.93±72.03	MS
**Ketones (5)**					
2-(4'-Chloro) styrylchromone	14.48	1002	ND	1,822.18±2,576.95	MS
Acetophenone	16.60	1071	10.63±15.04	119.97±63.45	MS
2-Nonanone	17.35	1093	ND	41.34±58.46	MS
1,4-Hexadecansultone	33.70	>1700	ND	15.22±21.52	MS
2,11-Dodecanedione	35.53	>1700	ND	13.01±18.39	MS

(1) RT: retention time. (2) RI: retention index. (3) I.D.: identification. (4) ND: not detected. (5) MS/RI: identification using both MS and RI. RI: identified using MS.
